# The Leicester Home for Neurasthenic Soldiers

**Published:** 1917-08-04

**Authors:** 


### THE VALUE OF VOLUNTARY EFFORT.

### The Leicester Home for Neurasthenic Soldiers.

The Mayor of Leicester (Alderman J. North, J.P.) was able to afford useful information as to the value which the Government Departments place upon voluntary effort, at a meeting held to appoint a Representative Committee to administer his £100,000 Fund for disabled sailors and soldiers. He expressed his appreciation of the liberal provision made by the Government, but stated that it would be found that there were exceptional cases which by no stretch of imagination could it be said were adequately recompensed for their sufferings and disabilities. It was just the provision for these exceptional needs that his Fund was formed to help. One felt want which had been suggested to him by the Government Departments was the need for a Home in the Midland Counties for the treatment of cases of neurasthenia arising from shock, from which a large majority of soldiers were suffering. There were already in existence two such Homes in England, and he hoped that Leicester would provide the third.

The Pensions Committee in London were anxious that such a Home should be established, and he at once thought that the present would be a very promising opportunity to acquire the Leicester Frith House, on the outskirts of Leicester, which was delightfully situated, and in his judgment a most suitable building at once available for the projected Home. He had accordingly made a tentative arrangement with the Estates Committee of the Town Council for the tenancy of the Home, and Sir John Collie, the Director of the National Neurasthenic Institution, had been deputed to report upon its possibilities. His report was altogether favourable and he had expressed the hope that Leicester would consent to provide such a Home. The Mayor therefore proposed for the Committee's consideration that the Leicester Frith House be acquired and furnished for the provision of 90 to 100 beds. They would then be asked to hand the Home over to the Pensions Committee, which would provide a Medical Officer, undertake the cost of maintenance, and be responsible to the wives and families, at the cost of the State, for pensions and allowances of the men receiving treatment. He regretted that the county had held aloof from the official support for his Fund, for he felt that inasmuch as county and borough had worked unitedly together in the matter of raising battalions and recruiting soldiers, who had fought side by side, those soldiers who had been maimed in the conflict but had escaped with their lives should have equal facilities for treatment. It was therefore his desire that everything in the shape of institutional treatment should be placed at the disposal of men in the county just as it was for men in the town; a sentiment which was cordially endorsed by the meeting.

### £62,500 Raised.

He (the Mayor) had hoped that the amount at which he aimed, £100,000, would have been received ere this, as it probably might have been had the effort been a joint one in town and county. The Mayor still hoped that even now the £100,000 would be forthcoming. Up to the present, the contributions totalled £62,432, of which £20,790 was raised at the recent Bazaar. £20,000 had been invested in War Loan and was producing a clear income of £1,000 per annum. It had been suggested to him that on his retiring from the office of Mayor, at the expiration of a three years' tenancy of that office, he should continue the chairmanship of the Fund. He had consulted the Mayor-Elect as to his wishes upon the matter and he had expressed his entire approval of the proposal. It was therefore probable that the name of the Fund would have to be changed to "The Disabled Heroes Fund" for Leicester, and he hoped that it would shortly be possible to add the words "and Leicestershire." Cordial approval of these proposals was expressed, and a committee was appointed to draw up a list of forty names, representative of all sections of the community, to form the Executive Committee.

